# Intraspecific and Geographical Variation of G*lossophaga commissarisi* in Mexico: Morphological Approach

**DOI:** 10.1093/iob/obag015

**Published:** 2026-04-30

**Authors:** D Vega-Montes de Oca, F Rodríguez-Gómez, C A Espinoza-Campuzano, M M Ramírez-Martínez, L I Iñiguez-Dávalos

**Affiliations:** Doctorado en Ciencias en Biosistemática, Ecología y Manejo de Recursos Naturales y Agrícolas de la Universidad de Guadalajara, Sede Centro Universitario de Ciencias Biológicas y Agropecuarias, Guadalajara, Jalisco 44600, México; Laboratorio de Análisis de la Biodiversidad y Genómica. Departamento de Bioingeniería Traslacional, Centro Universitario de Ciencias Exactas e Ingenierías, Universidad de Guadalajara, Guadalajara, Jalisco 44430, México; Laboratorio de Análisis de la Biodiversidad y Genómica. Departamento de Bioingeniería Traslacional, Centro Universitario de Ciencias Exactas e Ingenierías, Universidad de Guadalajara, Guadalajara, Jalisco 44430, México; Posgrado en Ciencias Biológicas, Facultad de Ciencias, Universidad Nacional Autónoma de México. Ciudad Universitaria, Coyoacán, Ciudad de México 04510, México; Departamento de Ciencias de la Salud y Ecología Humana, Centro Universitario de la Costa Sur, Universidad de Guadalajara, Autlán de Navarro, Jalisco 48900, México; Departamento de Ecología y Recursos Naturales, Centro Universitario de la Costa Sur, Universidad de Guadalajara, Autlán de Navarro, Jalisco 48900, México

## Abstract

Cryptic species represent a major challenge in biodiversity research due to their subtle and poorly defined morphological boundaries. This study investigates morphological variation within *Glossophaga commissarisi*, focusing on the differentiation between its two recognised subspecies, *G. c. commissarisi* and *G. c. hespera*, and evaluating the influence of biogeographic provinces, latitude, altitude, and sex. We applied geometric morphometrics (GM) to cranial structures and linear morphometric analysis (LM) to cranial and wing traits to assess patterns of variation. Significant shape differences were detected between subspecies, particularly in the fronto-maxillary region, independent of size, while the mandible showed size-dependent variation. Additionally, geographical factors, specifically elevation, latitude, and biogeographic province, emerged as key drivers of morphological divergence. Sexual dimorphism was evident in mandible and wing structures, with females displaying distinct mandibular and wing traits, potentially linked to reproductive and ecological roles. In *G. c. hespera*, geographic variation is noted in fronto-maxillary elevation; and by biogeographic provinces in ventral differentiation, with higher-altitude specimens differing from those of lowland populations in the Northern Pacific region. Similarly, *G. c. commissarisi* shows latitudinal variation, with Chiapas populations (16°N) distinct from those at 15°N and 17°N. These findings suggest that morphological differentiation in *G. commissarisi* may be better understood through biogeographic and ecological perspectives, complementing traditional morphological analyses. However, genetic and ecological data remain critical for clarifying taxonomic boundaries and evolutionary processes within these cryptic taxa. Limited sample availability due to the rarity of this species underscores the need for broader regional sampling in future research.

## Resumen

Las especies crípticas representan un desafío importante en la investigación de la biodiversidad debido a sus límites morfológicos sutiles y mal definidos. Este estudio investiga la variación morfológica dentro de *Glossophaga commissarisi*, centrándose en la diferenciación entre sus dos subespecies reconocidas, *G. c. commissarisi* y *G. c. hespera*, y evaluando la influencia de las provincias biogeográficas, la latitud, la altitud y el sexo. Aplicamos morfometría geométrica (GM) a las estructuras craneales y análisis morfométrico lineal (LM) a los rasgos craneales y alares para evaluar los patrones de variación. Se detectaron diferencias significativas en la forma entre subespecies, particularmente en la región fronto-maxilar, independientemente del tamaño, mientras que la mandíbula mostró una variación dependiente del tamaño. Además, los factores geográficos, específicamente la elevación, la latitud y la provincia biogeográfica, emergieron como impulsores en la divergencia morfológica. El dimorfismo sexual fue evidente en las estructuras de la mandíbula y el ala, con las hembras mostrando rasgos mandibulares y alares distintivos, potencialmente vinculados a roles reproductivos y ecológicos. En *G. c. hespera*, se observa variación geográfica en la elevación fronto-maxilar; y por provincias biogeográficas en la diferenciación ventral, con especímenes de mayor altitud que difieren de aquellos de las poblaciones de tierras bajas en la región del Pacífico Norte. De manera similar, *G. c. commissarisi* muestra variación latitudinal, con poblaciones de Chiapas (16°N) distintas de aquellas a 15°N y 17°N. Estos hallazgos sugieren que la diferenciación morfológica en *G. commissarisi* puede comprenderse mejor a través de perspectivas biogeográficas y ecológicas, complementando los análisis morfológicos tradicionales. Sin embargo, los datos genéticos y ecológicos siguen siendo importantes para aclarar los límites taxonómicos y los procesos evolutivos dentro de estos taxones crípticos. La disponibilidad limitada de muestras debido a la rareza de esta especie subraya la necesidad de un muestreo regional más amplio en futuras investigaciones.

## Introduction

Cryptic and pseudo-cryptic species refer to groups of taxa that are morphologically similar but can be distinguished by subtle genetic or morphological differences (Bickford et al. 2007; Fišer et al. 2018). This makes their identification and classification a challenging task. Even though two individuals may appear identical at first glance, they could belong to different species or subspecies (Bickford et al. 2007; Korshunova et al. 2019). Slight variations in the rostrum and cranium between lineages may result from selective pressures promoting specialization, a mechanism that reduces inter- or intraspecific competition and drives these morphological changes (López-Cuamatzi et al. 2024).

Several studies have emphasized that addressing the challenge of differentiating closely related species requires integrating diverse lines of evidence (Thorpe 1976; Hausser 1984; Polly 2003). Examining various sources of biological information can help distinguish taxa by identifying the sources of variation within and between populations (De Queiroz 2005; Srinivasulu et al. 2019). Morphometrics is particularly useful for this purpose, as it allows for a deeper analysis of morphological patterns influenced by the adaptive responses of traits, which are shaped by genetic control and the strength of selection acting upon them (Malhotra and Thorpe 1997; Radhekshmi and Polly 2005; López-Cuamatzi et al. 2024).

The nectar-feeding bats of the genus *Glossophaga* (long-tongued bats) exhibit strong morphological similarity, likely due to their closely resembling habitats, highly overlapping distributions, and their genetic similarity and/or phylogenetic affinities. *Glossophaga* includes nine species of Neotropical bats (Calahorra-Oliart 2022), most of which are primarily distinguished by cranial features and size (Webster 1993; Sánchez-Casas and Álvarez 2000). Their taxonomic history has been unclear and often confusing. Initially, these bats were categorized into two complexes, each containing multiple species and subspecies (Webster 1993).

The cryptic nature of *Glossophaga* species makes them one of the most challenging taxonomic bat complexes to identify. This challenge is further compounded by the fact that, even within the same species, morphological differences can be subtle or unclear (Webster 1993). Consequently, a more detailed examination of these taxa is required to understand their differentiation. This involves analyzing each taxon within the genus, including an investigation of intraspecific morphological variation.

Accordingly, this study focuses on elucidating the characteristics of *G. commissarisi* (Gardner 1962). In Mexico, the distribution of *G. commissarisi* includes two subspecies as follows: *G. commissarisi hespera*, in western Mexico, ranges from central Sinaloa to the states of Jalisco and Colima (Webster and Jones 1987). On the other hand, *G. commissarisi commissarisi* (Gardner 1962) distributes from the northwestern limit of its range in southern Veracruz and eastern Oaxaca to Chiapas and part of Campeche (southern Mexico, excluding the Yucatán Peninsula) (Webster and Jones 1980; Webster 1993). However, current distribution maps depict this species as continuously distributed throughout Mexico, from Sinaloa to Chiapas (Ceballos-González and Oliva 2005; Wilson et al. 2021; IUCN 2024; Burgin et al. 2020).

Following the original descriptions of both subspecies (*G. c. commissarisi* and *G. c. hespera*), main distinctions amongst them include *G. c. hespera* having a generally larger body and skull, a more vaulted basiocranial region, and a sharper rostral angle (Webster 1993), as well as their disjunct distribution (Webster and Jones 1987). The diagnostic reliability of these criteria is problematic, as there are no clear boundaries between the features and differences of the subspecies. The classification of lineages was based solely on fixed, observable morphological traits. However, these differences could reflect interpopulation phenotypic variation within the same species, incomplete lineage sorting, hybridization, or introgression (Tobias et al. 2010). Alternatively, they might represent distinct lineages resulting from geographical isolation.

Our study aims to use geometric morphometrics (GM) to analyze the morphological differences between the two subspecies, *G. c. commissarisi* and *G. c. hespera*. This technique enables the quantification of shape and size variations by analyzing anatomical structures through various data types, such as landmark coordinates, outline curves, and surfaces (Bookstein 1997). Geometric morphometrics is a powerful tool for examining shape variation and uncovering its underlying causes (Adams et al. 2013). Additionally, the analysis evaluated whether these morphological differences correspond to the biogeographic history of the species.

Although geometric morphometrics (GM) provides a highly sensitive framework for detecting subtle shape variation, we also included linear morphometrics (LM) to complement and corroborate the patterns detected by GM, an approach that is particularly relevant in cryptic taxa, where minor morphological differences can strongly influence evolutionary, functional, and taxonomic interpretations. In addition, LM enabled the inclusion of wing morphology. In museum specimens, wings cannot be fully extended or flattened without risk of damage, making GM impractical for capturing alar structures under standardized conditions. Finally, several biologically relevant traits in bats, including skull dimensions and wing elements, are closely linked to feeding performance, biomechanical function, and energetic demands; these traits are efficiently captured by linear measurements, facilitating direct comparisons with previous taxonomic, ecological, and functional studies (Nogueira et al. 2009).

## Materials and methods

### Data sources

We analyzed 66 specimens of the bat species *G. commissarisi*, representing two subspecies: 35 specimens of *G. c. commissarisi* and 31 specimens of *G. c. hespera* (supplementary Tables S1 and S2). The specimens were obtained from three mammal collections: Museo de Zoología “Alfonso L. Herrera” (MZFC) in the Sciences School, and Colección Nacional de Mamíferos (CNMA) in the Pabellón Nacional de la Biodiversidad, both housed at the Universidad Nacional Autónoma de México (UNAM); and Colección de Vertebrados (CV-DERN) of the Departamento de Ecología y Recursos Naturales (DERN), in the Centro Universitario de la Costa Sur (CUCSur), at the Universidad de Guadalajara (UDG). Several cranial features were used to confirm the identification of all analyzed specimens, including a shortened rostrum, reduced and evenly spaced lower incisors, and absent pterygoid wings (Álvarez-Castañeda et al. 1994; Sánchez-Hernández et al. 2016; Álvarez-Castañeda et al. 2017).

**Fig. 1:**
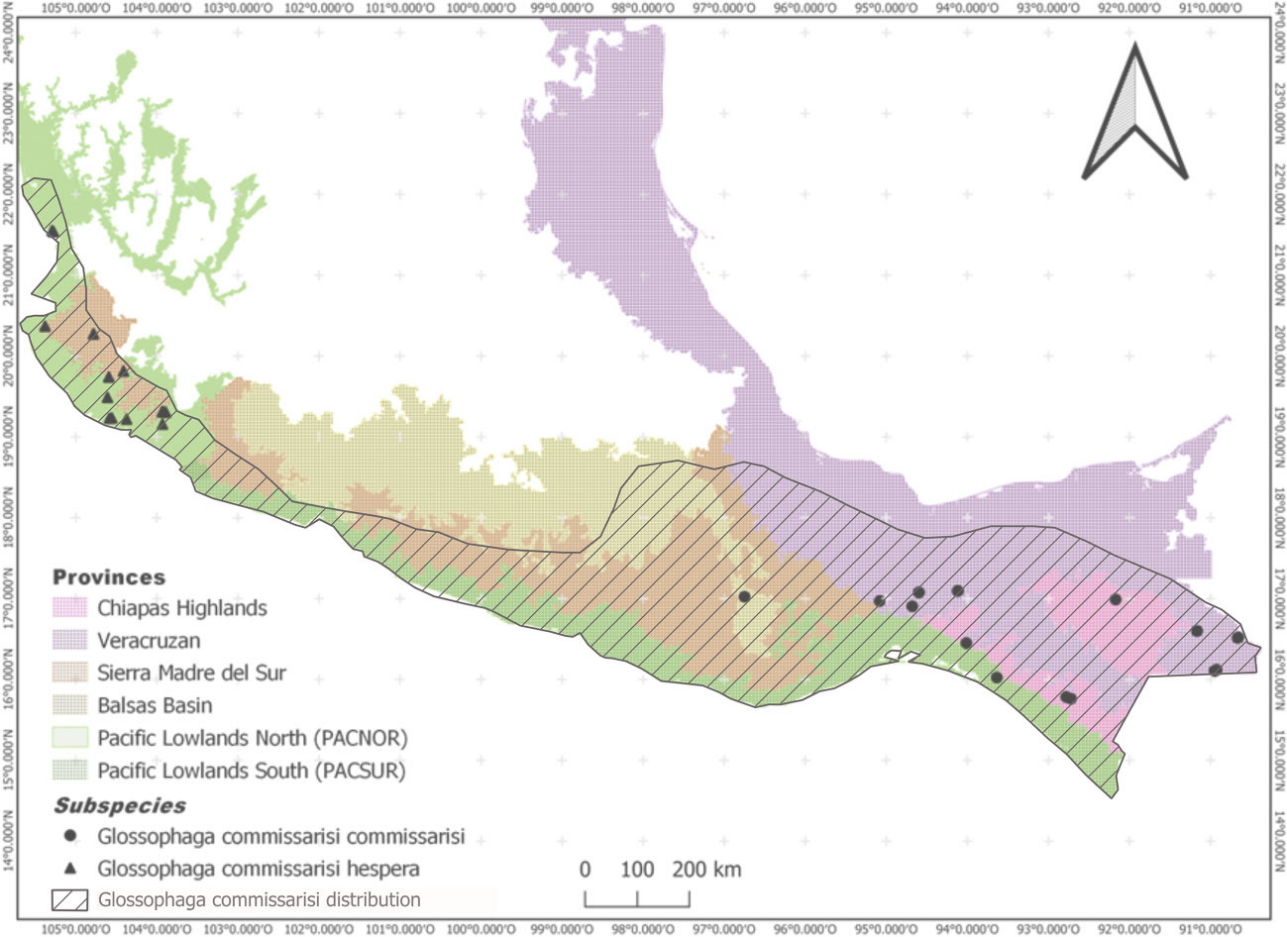
Map showing biogeographic provinces and localities where specimens were originally collected in Mexico.

The analyzed specimens originate from various Mexican biogeographic provinces (Morrone et al. 2017): Chiapas Highlands (CH), Sierra Madre del Sur (SMS), Veracruz Province (VP), Balsas Basin (BB), and the Pacific Lowlands, which are further divided into southern (PACSUR) and northern (PACNOR) regions (Fig. 1). The specimens were collected along an elevation gradient ranging from 2 meters above sea level (m.a.s.l.) to 1,557 m.a.s.l., and within a latitudinal range from 21.553920°N (Nayarit, Mexico) to 15.4544°N (Chiapas, Mexico). Some of these specimens were included in either linear morphometric analyses, geometric morphometric analyses, or both.

### Geometric morphometric (GM)

#### Sample collection and data acquisition

Specimens from mammal collections were photographed in manual mode using a Nikon Coolpix P900 camera (Nikon Co., Tokyo, Japan) with a macro focus setting. Digital photographs were taken of the mandibles and skulls, ensuring consistent positioning for lateral and ventral views. A 1 cm scale was included in each image for reference.

#### Landmark system

**Fig. 2:**
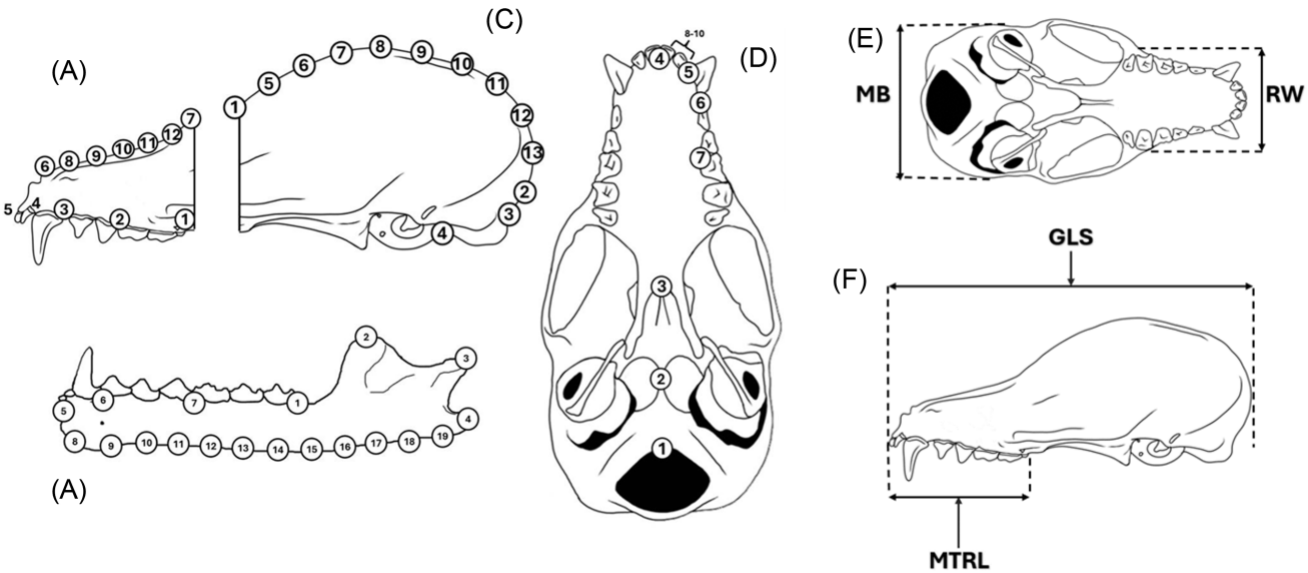
Landmark configurations used for geometric morphometric analyses across four views of the skull: (A) fronto-maxillary view, (B) mandible, (C) parieto-occipital view, and (D) ventral view of the cranium. Panels (E–F) illustrate traditional cranial linear measurements recorded for this study, including mastoid breadth (MB), rostrum width (RW), greatest length of skull (GLS), and maxillary toothrow length (MTRL).

Four two-dimensional landmark configurations were digitized to examine variation in *Glossophaga commissarisi* (Fig. 2, supplementary Table S3) using the software tpsUtil and tpsDig2 (Rohlf 2017; Rohlf 2019). Semilandmarks were positioned by equidistantly dividing the curves traced along the outlines of specific structures (Ospina-Garcés and León-Paniagua 2021).

To assess variation, we divided the lateral view of all skulls into two regions: fronto-maxillary and parieto-occipital. These regions correspond to distinct developmental areas (rostral and basicranial) identified in previous studies (Goswami 2006; Marroig et al. 2009; Porto et al. 2009). Additionally, we analyzed the ventral side of cranium, and the mandible lateral view. Each view was examined using a specific number of landmarks (supplementary Table S3). Most of these landmarks were previously used to study *Glossophaga soricina* (Calahorra-Oliart et al. 2021). However, some landmarks were excluded due to the lack of homology across all sampled specimens.

To remove the effects of scale, position, and orientation on the landmark data, a Generalized Procrustes Analysis (GPA) was performed using the gpagen function from the R package geomorph (Adams et al. 2025; Velazco et al. 2023). In addition to the Procrustes shape coordinates, we extracted a size metric known as centroid size (Cs). Centroid size is calculated as the square root of the sum of the squared distances between each landmark and the geometric center (centroid) of the entire landmark configuration (Bookstein 1997).

Measurement error was assessed following the procedures recommended by Cardini (2024a, 2024b, 2025), which evaluate landmark digitizing precision through repeated digitizations and variance partitioning using Procrustes ANOVA and Measurement Error (ME) ANOVA. This method compares two independent digitizations of the same specimens (or a representative subset) to test whether variation among replicates is significant relative to true biological variation. Each cranial configuration was digitized twice under identical conditions, generating replicate datasets (v_repetition). A two-factor Procrustes ANOVA was then conducted with:

v_discrete: variation among individuals (biological source)

v_repetition: variation among replicates (digitizing error)

The analysis was based on 10,000 permutations to compute p-values for F and Z statistics, determining the significance and magnitude of measurement error (Table S5).

### Statistical models

To evaluate morphological variation among subspecies and the influence of various factors, we performed Procrustes ANOVA and linear distance-based models using the R package (version 4.0.9; Adams et al. 2024). These models tested the effects of subspecies (Subsp), sex nested within subspecies (sex), biogeographic provinces (bp), elevation (elev), and latitude (lat). Continuous variables were log-transformed prior to analysis. Two separate models were applied to distinguish the effects of shape and size on morphological variation.

#### Model 1

This model focused exclusively on shape variation by removing the influence of size through a two-step process. First, allometry was assessed using a linear model of shape versus size. Allometry refers to the size-related changes in morphological traits (Klingenberg 2016). Residuals from this model were extracted and adjusted by adding the sample mean, effectively isolating the shape component, independent of size. The resulting adjusted shape data were then subjected to Procrustes ANOVA. This approach follows the Gould-Mosimann allometric framework (Gould 1966; Mosimann 1970; Klingenberg 2016), which explores the correlation between size and shape. This approach is a widely used method for removing the allometric component (or size influence) of shape variation in morphometric research (Rosas and Bastir 2002; Weisensee and Jantz 2011; Ponssa and Candioti 2012).

#### Model 2

Raw coordinate model. Size is a significant determinant of variation in many organismal traits. This model utilized raw landmark data (coordinates), preserving the influence of size. Centroid size (Cs) was included as a factor to evaluate its contribution to overall variation, alongside the other factors analyzed in the previous model.

### Ordination methods and shape change visualizations

Multivariate patterns of cranial and mandibular shape variation were examined using Principal Component Analysis (PCA) applied to the Procrustes-aligned coordinates. PCA was conducted with gm.prcomp from the geomorph package v4.0.5 (Adams and Otárola-Castillo 2013; Adams et al. 2025) in R v4.3.2 (R Core Team 2022). Individual scores on the first two principal components (PC1–PC2) were plotted to visualize specimen distribution in morphospace by subspecies and sex. Scatterplots generated using base R graphics and ggplot2 v3.4.4 (Wickham 2009) were used to assess clustering, intergroup overlap, and within-group dispersion.

Shape changes along the principal axes and across ecological and biological gradients (latitude, elevation, and biogeographic provinces) were visualized using vector-based deformation plots. Mean→Max and Mean→Min transformations for PC1 and PC2 were computed and displayed with plotRefToTarget(method = “vector”) (geomorph v4.0.5). Vector length was magnified 3 × to aid interpretation, with arrows indicating the direction and magnitude of landmark displacement relative to the mean configuration.

Differences in centroid size (Csize) across subspecies, sexes, and ecological categories were illustrated using boxplots (ggplot2 v3.4.4), allowing visualization of size divergence and sexual dimorphism. Landmark-specific variability was further summarized by computing Procrustes displacement per landmark and displaying the results using barplots, enabling identification of regions with the highest morphological variation (Zelditch et al. 2012).

### Modularity and integration

To evaluate the correlation between the two side-view modules of the cranium (maxilla and parieto-occipital), a Partial Least Squares (PLS) analysis was performed in the context of Integration and Modularity (2B-PLS). The statistical significance of these correlations was determined through permutation tests with 1000 random repetitions utilizing the R package “geomorph” version 4.0.9 (Adams et al. 2024).

### Linear morphometrics (LM)

Analyses abbreviations: **LM** = Linear Morphometrics, **GM** = Geometric Morphometrics, **LCA** = Linear Cranial Analysis, **LWA** = Linear Wing Analysis. Specimens for the Linear Morphometrics (LM) analysis were measured using a Mitutoyo CD-6" caliper (Mitutoyo U.S.A.) with a precision of ± 0.1 mm. To minimize bias and ensure consistency, all measurements were taken by the same person. These linear measurements were divided into two subcategories. The LWA analysis includes forearm (FA), thumb (TH), metacarpal in the third and fourth digit (M3, M4), first and second phalanges in digit three (P1-D3, P2-D3), and first phalanx from digit four: P1-D4. The LCA included greatest length of the skull (GLS), maxillary toothrow length (MTRL), mastoid breadth (MB) (Garbino et al. 2024), and rostrum width (RW).

For the statistical analysis, a non-parametric approach was applied using permutational multivariate analysis of variance (PERMANOVA) on square root-transformed data using a Bray-Curtis similarity matrix. P-values were calculated using 9,999 permutations based on a sequential model and Type I sum of squares. When significant values (*p* < 0.05) were identified, post-hoc permutational T-tests were conducted to assess pairwise differences.

In both, LCA and LWA, sex was included as a factor in the analyses, as females are documented to be slightly larger than males in several bat species (Wu et al. 2018; Maucieri et al. 2021; Castillo-Figueroa 2024). Sex was nested within each lineage. Other factors included in the analysis were biogeographic province, altitude, and latitude. Finally, Principal Coordinate Analysis (PCoA) was performed to visualize and group the analyzed factors.

## Results

### Fronto-maxillary region

#### Conspecific differentiation (between subspecies)

**Fig. 3:**
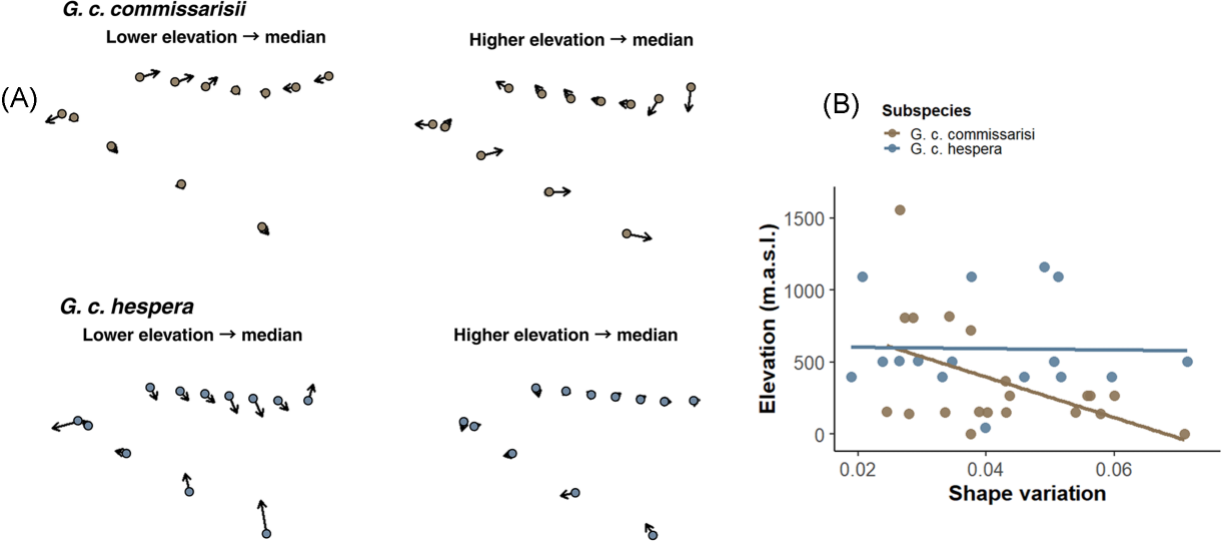
Fronto-maxillary shape variation and elevation. Left: Lollipop plots comparing median fronto-maxillary shape with low- and high-elevation specimens for each subspecies. Right: Bivariate plot showing the relationship between elevation and fronto-maxillary shape variation (Procrustes distance) for both subspecies.

Significant shape differences were detected between subspecies in the fronto-maxillary region (Model 1, r² = 0.090, *p* = 0.010). These differences persisted even after accounting for size (Model 2, r² = 0.088, *p* = 0.008; Table 1), while size itself was not a significant factor (Model 2, r² = 0.023, *p* = 0.469,). This indicates that shape, rather than size, drives variation in this region, with changes primarily concentrated on the rostral slope of the maxilla (Fig. 3a).

**Table 1:** Procrustes analysis of variance (ANOVA) for geometric morphometrics

			df	*R^2^*	*F*	*P value*
**Fronto-maxillary**	**Shape and form (Model 1)**	**Subspecies**	1	0.0901	3.535	0.010*
		**Elevation(masl)**	1	0.0897	3.5197	0.005**
		**BP**	3	0.0534	0.6991	0.761
		**Latitude**	1	0.0233	0.9139	0.456
		**sex (sub)**	2	0.5494	1.0773	0.388
		**Residuals**	27	0.6884		
	**Allometry (Model 2)**	**Csize**	1	0.023	0.8991	0.469
		**Subspecies**	1	0.0883	3.4506	0.008**
		**Elevation(masl)**	1	0.0877	3.4245	0.005**
		**BP**	3	0.0534	0.6956	0.759
		**Latitude**	2	0.0261	1.0211	0.4
		**sex (sub)**	26	0.0555	1.0839	0.387
		**Residuals**	35	0.6658		
**Mandible**	**Shape and form (Model 1)**	**Subspecies**	1	0.1032	3.648	0.004**
		**Elevation(masl)**	1	0.0478	1.687	0.102
		**BP**	3	0.1348	1.585	0.056
		**Latitude**	1	0.0148	0.525	0.857
		**sex (sub)**	2	0.1323	2.335	0.009**
		**Residuals**	28	0.5668		
	**Allometry (Model 2)**	**Csize**	1	0.1005	3.0692	0.001 ***
		**Subspecies**	1	0.0985	2.9757	0.004 **
		**Elevation(masl)**	1	0.0440	1.2461	0.112
		**BP**	3	0.1246	1.5091	0.064 .
		**Latitude**	1	0.0118	−1.3040	0.906
		**sex (sub)**	2	0.1202	2.3958	0.010 **
		**Residuals**	28	0.5005		
**Ventral**	**Shape and form (Model 1)**	**Subspecies**	1	0.0206	0.6499	0.688
		**Elevation(masl)**	1	0.0141	0.4435	0.861
		**BP**	2	0.1197	1.8800	0.045*
		**Latitude**	1	0.0200	0.6291	0.72
		**sex (sub)**	2	0.0931	1.4627	0.151
		**Residuals**	23	0.7322		
	**Allometry (Model 2)**	**Csize**	1	0.0921	3.1684	0.008**
		**Subspecies**	1	0.0217	0.7482	0.619
		**Elevation(masl)**	1	0.0138	0.4729	0.823
		**BP**	2	0.1230	2.1143	0.025*
		**Latitude**	1	0.0252	0.8686	0.51
		**sex (sub)**	2	0.0840	1.4443	0.143
		**Residuals**	22	0.6400		
**Parieto-occipital**	**Shape and form (Model 1)**	**Subspecies**	1	0.03511	1.0522	0.401
		**Elevation(masl)**	1	0.01622	0.4859	0.786
		**BP**	3	0.11901	1.1888	0.281
		**Latitude**	1	0.09254	2.7732	0.021*
		**sex (sub)**	2	0.03633	0.5444	0.871
		**Residuals**	21	0.70079		
	**Allometry (Model 2)**	**Csize**	1	0.08547	2.7869	0.02*
		**Subspecies**	1	0.03854	1.2566	0.287
		**Elevation(masl)**	1	0.02106	0.6867	0.631
		**BP**	3	0.12512	1.36	0.187
		**Latitude**	1	0.08169	2.6638	0.022*
		**sex (sub)**	2	0.03479	0.5673	0.846
		**Residuals**	20	0.61334		

Landmark configurations include the fronto-maxillary region, mandible, ventral skull view, and parieto-occipital region. Model 1 represents the shape model; Model 2 incorporates allometry to model. BP = Biogeographical provinces * P < 0.05; ** P < 0.01.

### Elevation-related variation

Although elevation was statistically significant, the associated effect size was small (R² ≈ 0.09 in both models), indicating that only a modest proportion of maxillary shape variation is attributable to altitudinal differences. The lollipop plots comparing low- and high-elevation specimens (Fig. 3a) show localized landmark displacements in both subspecies, indicating subtle maxillary shape differences along the elevational gradient. Consistently, the correlation of shape variation against elevation (Fig. 3b) reveals a shallow trend in *G. c. hespera* and virtually no association in *G. c. commissarisi*. These visual patterns confirm that elevation is statistically significant, and its effect on maxillary shape is relatively modest.

These results indicate that fronto-maxillary shape variation is predominantly explained by subspecies identity and elevational differences, whereas the contributions of geographic province, latitude, and sex are statistically non-significant.

### Mandible

#### Conspecific differentiation

**Fig. 4:**
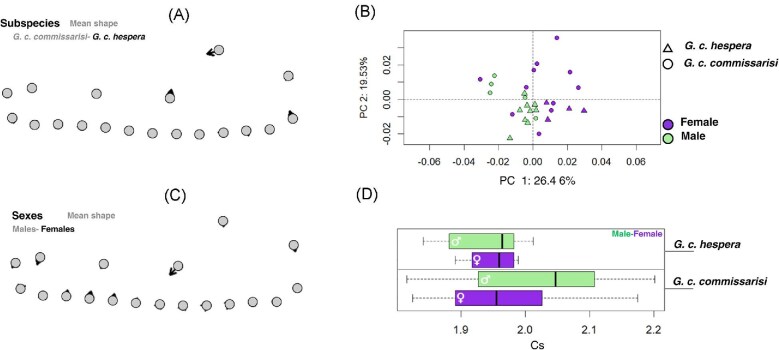
Mandibular variation analyzed using geometric morphometrics (GM). Sexes and subspecies. (A) Mean-shape differences between subspecies (*G. c. commissarisi* and *G. c. hespera*). (B) Mean-shape contrasts between males and females. (C) Principal Component Analysis (PCA) of mandibular shape showing subspecies and sexes. (D) Centroid size (Cs) boxplot variation by sex within each subspecies.

Procrustes ANOVA indicates a statistically significant subspecies effect (Model 1: R² = 0.103, *p* = 0.004; Table 1), the PCA shows extensive overlap between lineages and sexes (Fig. 4b). PC1 accounted for 26.5% of the total variance and PC2 for 19.5%, jointly explaining 46.0% of the variation (supplementary  Table S4). Vector-based comparisons (Fig. 4a, supplementary  Fig. S3a-d) and landmark displacement barplots (supplementary  Fig. S3e) reveal that mandibular variation is concentrated in the coronoid and angular regions, which exhibit the strongest, but still subtle landmark shifts across individuals. The displacement vectors are short, indicating that mandibular architecture is largely conserved and does not show clear group-specific signatures.

#### Sexual dimorphism

Sex nested within subspecies showed significant differences in mandibular shape (Model 1, r² = 0.132, *p* = 0.009; Model 2, r² = 0.120, *p* = 0.010; Table 1), indicating detectable but subtle sexual dimorphism. Mean-shape comparisons (Fig. 4c) revealed that females exhibit a slightly more pronounced curvature of the coronoid process. In the PCA morphospace (Fig. 4b), males and females overlap broadly, and females tend to occupy higher PC1 values, while males display greater dispersion across both axes. Centroid size patterns (Fig. 4d) are slightly larger averages in females and greater size variability in *G. c. commissarisi*.

These results indicate that mandible shape variation is explained mainly by subspecies identity and sexual dimorphism, whereas the contributions of geographic province, latitude, and elevation are statistically non-significant in the models explored.

### Ventral view of skull

#### Biogeographic province

The ventral region exhibited significant shape differences among biogeographic provinces (Model 1, r² = 0.119, *p* = 0.045; Model 2, r² = 0.123, *p* = 0.025; Table 1), and centroid size also varied significantly (r² = 0.092, *p* = 0.008). Post hoc t-tests indicated significant differentiation between populations from the Northern Pacific Lowlands (PACNOR) and the Chiapas Highlands (CHH) (d = 0.019, *p* = 0.001), and between the Chiapas Highlands and the Veracruz Province (d = 0.015, *p* = 0.0027).

**Fig. 5:**
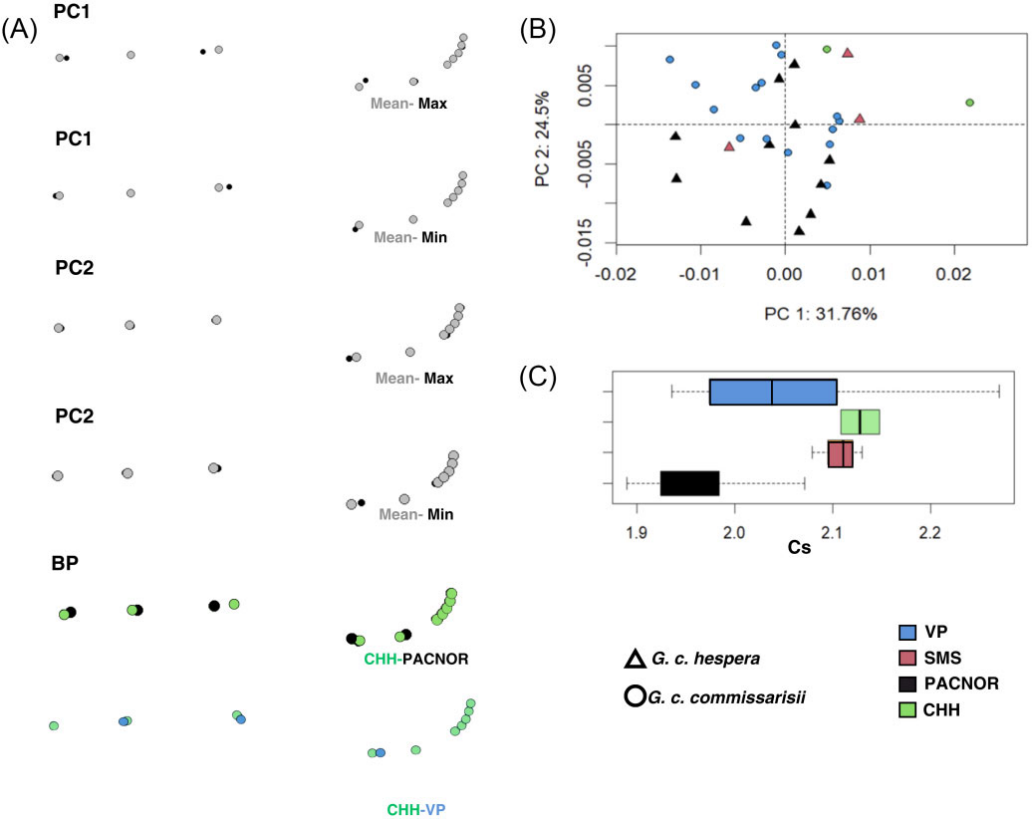
Ventral skull configuration analyzed using geometric morphometrics (GM). (A) Shape changes along PC1 and PC2, Biogeographic provinces (BP) illustrated with points method. (B) Principal Component Analysis including centroid size (Cs). (C) Boxplot of Cs and biogeographic provinces. A 3x magnification was included.

In the ordination plot (Fig. 5b), PC1 explained 31.8% of variance and PC2 explained 24.5%, together accounting for 56.3% of total variation (supplementary Table S4). The spatial distribution shows a partial separation between *G. c. hespera* (triangles) and *G. c. commissarisi* (circles), with *hespera* tending toward positive PC1 values and *commissarisi* clustering near the origin.

PC2 reflects geographic differentiation: CHH individuals occupy more positive PC2 values, whereas VP and SMS show greater dispersion and overlap. Although overlap is present, the pattern reveals spatial tendencies associated with both subspecies and biogeographic province.

In PC1, the greatest deformation occurs along the palatal region (Fig. 5a), where landmarks associated with the palatine suture (Landmark 3, Fig. 2d) exhibit the largest displacements, indicating changes in palatal width and length. The Mean–Max and Mean–Min extremes show that PC1 primarily represents a gradient from narrower, elongated palates to wider ones.

The Biogeographic Province panel of Fig. 5 shows that CHH–PACNOR comparisons display larger landmark displacements than CHH–VP, indicating stronger geographic divergence in ventral cranial morphology in the former comparison.

The centroid-size boxplot (Fig. 5c) reveals geographic differences in ventral cranial size. CHH shows the highest Cs values, indicating larger or more robust ventral crania, whereas VP and PACNOR exhibit intermediate values with overlapping ranges. This pattern suggests significant geographic variation in size, consistent with the differences observed in shape and landmark displacement.

Other factors (Altitude, Latitude, and Sex) were not statistically significant in explaining variation in the ventral cranial landmark configuration.

### Parieto-occipital view of skull

#### Latitude variation

Latitude showed a significant effect on parieto-occipital shape in both the shape-only model (Model 1: R² = 0.09254, F = 2.7732, *p* = 0.021) and the allometric model (Model 2: R² = 0.08169, *p* = 0.022; Table 1). Centroid size was also significant in the allometric model (R² = 0.08547, *p* = 0.02). These results indicate consistent but subtle latitudinal structuring of parieto-occipital morphology.

**Fig. 6:**
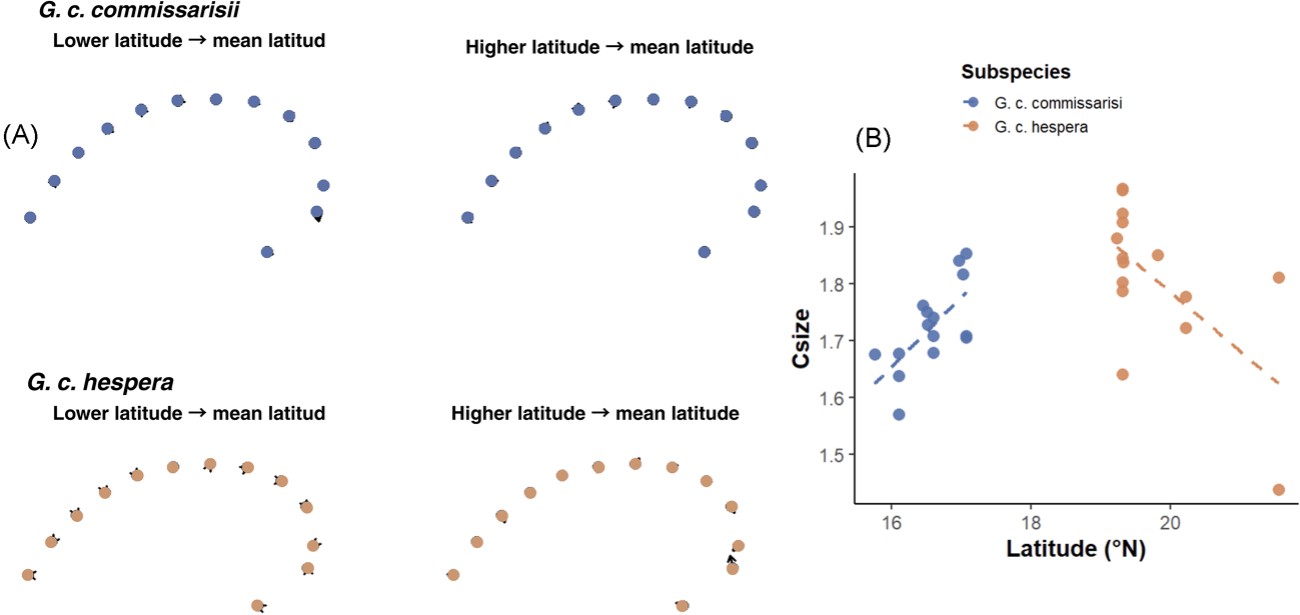
Parieto-occipital skull configuration analyzed using geometric morphometrics (GM). (A) Lollipop plots showing landmark displacement between lower- and higher-latitude specimens in each subspecies, with vectors magnified 3 × . (B) Relation between centroid size (Cs) and latitude, illustrating allometric trends in *G. c. commissarisi* and *G. c. hespera.*

Lollipop plots show short landmark displacements across latitude in both subspecies (Fig. 6a). In *G. c. commissarisi*, shifts occur mainly along the lateral margins and posterior arch (Landmark number 2 in this configuration), whereas *G. c. hespera* exhibits similarly subtle adjustments along the parietal curvature. Centroid size trends differ between subspecies (Fig. 6b): *G. c. commissarisi* increases slightly with latitude, while *G. c. hespera* decreases. Overall, parieto-occipital shape and size patterns reflect subtle but geographically structured latitudinal variation.

#### Modularity

The cranial lateral views (fronto-maxillary and parieto-occipital) displayed low but significant integration (r = 0.707, *p* = 0.02). Furthermore, the significant covariation ratio (CR = 0.87, *p* = 0.003) supports this modular organization. A CR value between 0 and 1 indicates that these structures covary less with each other than within their respective modules, suggesting functional and developmental independence between the cranial modules.

### Linear morphometrics

#### Subspecies differences

**Fig. 7.**
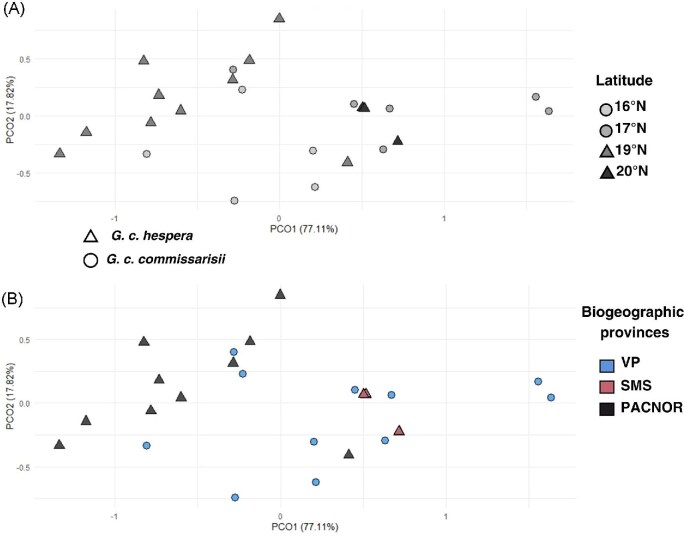
The Principal Coordinate Analysis (PCoA) of the linear cranial analysis (LCA). (a) Visualization of latitude in the morphospace. (b) Visualization of biogeographic provinces in the morphospace.

Summary statistics and boxplots (Table 2, supplementary  Fig. S1) showed that *G. c. commissarisi* tends to be slightly larger, particularly in the greatest length of the skull (GLS), rostral width (RW), and mastoid breadth (MB). Additionally, *G. c. commissarisi* exhibited greater variability in these measurements compared to *G. c. hespera.* Linear Cranial Analysis (LCA) revealed significant differences between the two subspecies (r² = 0.38, *p* = 0.0001; Table 2). Most *G. c. hespera* individuals clustered on the left side of the PCoA plot, corresponding to negative PC1 values, whereas *G. c. commissarisi* individuals were more dispersed across the plot (Fig. 7).

**Table 2:** Permutational multivariate analysis of variance (PERMANOVA) of linear cranial analysis (LCA) and linear wing analysis (LWA).

		PERMANOVA		
		**Factor**	**Sq. root**	***p*-value**
**LCA**		**Bp**	0.73	0.0009***
		**Subsp**	0.38	0.0001***
		**Elevation**	−0.18	0.7567
		**Lat**	0.77	0.003**
		**Sex (sub)**	0.26	0.12
		**Residuals**	0.65	
**LWA**		**Bp**	0.295	0.1430
		**Subsp**	0.133	0.3330
		**Elevation**	0.265	0.0720
		**Latitude**	0.854	0.0001***
		**Sex (sub)**	0.362	0.018*
		**Residuals**	1.019	
**PAIRWISE TESTS**				
**LCA**	***G. c. hespera***	**Bp**	PACNOR-SMS	*p* = 0.0003***
	***G. c. commissarisi***	**Lat**	16°N-17°N	*p* = 0.01*
**LWA**	***G. c. commissarisi***	**Lat**	16°N-15°N	*p* = 0.0151*
			16°N-17°N	*p* = 0.0001***

Post-hoc tests of biogeographic provinces (BP) and latitude (Lat). * *p* < 0.05; ** *p* < 0.01; *** *p* < 0.001. Subspecies (subsp), sex nested within subspecies (sex), biogeographic provinces (bp), elevation (elev), and latitude (lat).

#### Variation across Mexican biogeographic provinces

The LCA analysis identified significant differences among biogeographic provinces (r² = 0.73, *p* = 0.0009; Table 2). Post-hoc tests revealed significant variation between the PACNOR and the Sierra Madre del Sur (SMS) provinces (t = 2.84, *p* = 0.0003).

Principal Coordinates Analysis (PCoA) showed that nearly all individuals from VP are grouped in the negative section of the PCO2 axis, while most PACNOR individuals are primarily distributed throughout the negative portion of the PCO1 axis. Bats from the SMS province (*G. c. hespera* subspecies) tend to cluster with those from VP (*G. c. commissarisi* subspecies), as illustrated by the biogeographic provinces PCoA (Fig. 7-B) and further supported by cranial measurement boxplots (supplementary Fig. S1).

#### Latitude variation

Latitude had a significant influence on LCA differentiation (r² = 0.77, *p* = 0.003; Table 2). Pairwise comparisons revealed significant differences between individuals at 16°N and 17°N latitudes (t = 2.50, *p* = 0.01; Table 2). In the ordination plot, individuals at 16°N exhibited lower, negative PCO2 values, whereas those at 17°N had positive and higher PCO1 values. Boxplots (supplementary Fig. S1) visually illustrate size differences between these latitude groups.

In LWA, significant differences were observed (r² = 0.854, *p* = 0.0001) between individuals at 16°N and 15°N (t = 1.93, *p* = 0.015, Table 2) and between 16°N and 17°N (t = 2.93, *p* = 0.0001). Individuals at 17°N had larger TH, M4, and M3 measurements compared to other latitudes (supplementary Fig. S2).

### Sexual Dimorphism

Significant differences between males and females were observed (*p* = 0.018, r² = 0.362; Table 2). In the subspecies *G. c. commissarisi*, females tended to be larger in FA, TH, M3, and M4. However, no consistent patterns of sexual dimorphism were detected in *G. c. hespera* in the boxplots (supplementary Fig. S2).

## Discussion

### Cryptic diversity and the challenge of species delimitation

One of the current challenges in biological sciences is understanding cryptic diversity, particularly when empirical evidence increasingly shows that morphological similarity may conceal substantial evolutionary divergence, a topic with important implications for the recognition of species and evolutionary lineages (Bickford et al. 2007; Pfenninger and Schwenk 2007). A major obstacle to studying cryptic diversity arises from the multiple species concept and the lack of consensus on how species should be accurately represented and delimited (Fišer et al. 2018; Struck et al. 2018), a difficulty that becomes especially evident in groups where phenotypic differentiation is subtle, continuous, or geographically structured rather than discrete.

Previous molecular studies have provided an essential phylogenetic framework for understanding diversification within *Glossophaga*, consistently recovering *Glossophaga soricina* as the basal sister lineage of the genus (Hoffmann and Baker 2001). These foundational phylogenetic hypotheses have since guided expectations regarding patterns of divergence within the genus, and subsequent integrative approaches combining molecular and morphological data have demonstrated that cryptic diversity is widespread within *Glossophaga*, revealing deeply divergent lineages within *G. soricina* that are not readily distinguishable using traditional morphological characters alone (Calahorra-Oliart et al. 2021). Together, these studies highlight that morphological conservatism does not necessarily reflect evolutionary homogeneity within the genus.

This study contributes to this field by examining morphological diversity in *Glossophaga commissarisi* and its two subspecies in northern Mexico. While our findings support some distinctions previously described for these subspecies (Webster 1993), the incorporation of multiple spatial and ecological axes reveals a more complex pattern of intraspecific variation than previously recognized, as additional factors such as biogeographic provinces, altitude, and latitude uncover structured morphological differentiation. These results suggest that morphological variation in *G. commissarisi* is not random, but instead reflects geographically mediated processes, pointing to a more complex taxonomic scenario. A more precise classification will require further investigation, particularly because phenotypic divergence does not always correspond to genotypic divergence (Schmid and Guillaume 2017), a pattern that has been repeatedly documented in cryptic and pseudo-cryptic taxa.

### Intraspecific morphological variation in *Glossophaga commissarisi*

Geometric morphometric analyses detected differences between *G. c. hespera* and *G. c. commissarisi* in two out of four landmark configurations: the fronto-maxillary region (shape differences) and the mandible (size and shape differences). Variation in the fronto-maxillary region was characterised by differences in the angle between the rostrum and cranium (rostral slope), a feature noted in previous descriptions of the subspecies (Webster 1993). This angle varies markedly among *Glossophaga* species and has been associated with nectarivory levels (Freeman 1995; Solmsen 1998; Dumont 2004; Quinche et al. 2023).

The maxillary shape variation identified here contrasts with patterns reported for *Glossophaga mutica* and *G. valens*, which exhibit identical fronto-maxillary shapes despite being genetically and morphologically distinct (Calahorra-Oliart et al. 2021). However, because the functional relevance of the rostral slope remains uncertain, additional research is needed to determine whether it reflects anatomical integration or ecological adaptation. For the mandible, the main axis of variation occurred in the coronoid process, an anatomical structure associated with temporalis muscle strength. A larger coronoid process increases temporalis contraction capacity, enhancing bite force (Solmsen 1998). Conversely, a reduced coronoid process has been linked to enhanced tongue support in nectarivorous bats at the expense of bite force (Nogueira et al. 2009).

Linear cranial measurements also showed a trend of slightly larger dimensions in *G. c. commissarisi*, especially GLS, RW, and MB (Table 3; supplementary Fig. S1). Similar patterns occur in the *Artibeus lituratus* cryptic complex, where subspecies differ in body size and skull robustness according to regional precipitation regimes (Marchán-Rivadeneira et al. 2011).

**Table 3:** Summary of cranial and wing measurements between subspecies of *Glossophaga commissarisi*.

MEASURE (mm)	*G. c. commissarisi*	*G. c. hespera*
**LINEAR CRANIAL ANALYSIS (LCA)**		
**GLS**	20.55 ± 0.51(19.78–21.47) 12	20.35 ± 0.51(19–20.97) 14
**MTRL**	8.00 ± 0.43(7.3–9)12	8.01 ± 0.50(7.2–9.2)14
**RW**	6.14 ± 0.22(5.8–6.5)12	5.89 ± 0.25(5.6–6.3)14
**MB**	9.76 ± 0.38(9.2–10.7)12	9.36 ± 0.26(9–9.9)14
**LINEAR WING ANALYSIS (LWA)**		
**AB**	34.77 ± 1.23(31.5–38.3)33	35.84 ± 0.84(34.58–37.76)27
**TH**	7.40 ± 0.91(5.46–8.99)33	7.16 ± 0.72(5.75–8.54)27
**M3**	32.29 ± 1.99(29.2–36.5)33	31.96 ± 1.55(28.85–36.47)27
**F1-D3**	12.89 ± 0.68(11.71–15.1)33	13.21 ± 0.56(12.24–14.81)27
**F2-D3**	15.04 ± 0.67(13.8–14.7)33	15.05 ± 0.70(13.6–17.05)27
**M4**	30 ± 1.91(27.32–33.84)33	29.58 ± 1.45(27.38–33.73)27
**F1-D4**	9.86 ± 0.72(8.18–12.29)33	10.16 ± 0.55(9.28–11.62)27

Overall, morphological differences between the subspecies are concentrated in the rostral region, a key anatomical module for feeding, consistent with dietary specialization patterns documented across phyllostomid bats (Freeman 2000; Van Cakenberghe et al. 2002; Hedrick and Dumont 2018).

### Functional and ecological interpretation

Several results point toward functional differentiation between subspecies. *G. c. commissarisi*, also distributed in Caribbean lowland forests of Costa Rica, is considered a relatively generalist flower-visiting bat with high dietary flexibility, supplementing nectar with insects and fruits (Bechler et al. 2024). A broader diet likely requires greater bite force than a strictly nectar-based diet, consistent with the relatively larger mandibular structures observed in this subspecies (Nogueira et al. 2009).

Maxillary and mandibular variation may therefore reflect differences in feeding ecology, anatomical integration, or niche breadth. However, future functional analyses will be required to clarify the ecological roles of traits such as rostral slope and coronoid height.

### Geographic variation

#### Geographic variation in *G. c. hespera*

The combination of elevation and biogeographic differentiation may explain the observed variation within *G. c. hespera*. Populations from higher altitudes (SMS province) differ from those at lower altitudes (PACNOR), as shown by GM analyses of the fronto-maxillary configuration and by LCA comparisons of linear cranial measurements.

LCA results revealed that PACNOR individuals differed from those in the northern sector of the SMS province, despite both belonging to *G. c. hespera* (Fig. 7b). The SMS province lies in the Mesoamerican highlands, part of the Mexican Transition Zone, that is a region marked by discontinuous species ranges and geographic barriers that often limit gene flow and promote differentiation (Morrone et al. 2017; Rocha-Méndez et al. 2019). Similar processes have driven divergence in bats (Hernández-Canchola and León-Paniagua 2017), birds (Rocha-Méndez et al. 2019), and other vertebrates (Bradley et al. 2004; Reyes-Velasco et al. 2015).

In both subspecies, the fronto-maxillary region exhibited the most pronounced landmark displacement between low- and high-elevation individuals. Although absolute differences were small—as expected in cryptic taxa—the direction of shape change was consistent within each subspecies, suggesting that even subtle elevational differences can induce detectable morphological responses.

Comparable elevational patterns have been documented in other nectarivorous and frugivorous bats (e.g., *Carollia brevicauda, C. perspicillata*; López-Aguirre et al. 2015). In contrast to classic highland–lowland divergences where strong cranial reorganization occurs, *Glossophaga commissarisi* exhibits more localized adjustments, primarily affecting rostral curvature and projection.

Likewise, elevation-driven morphological divergence has resulted in taxonomic differentiation in other bat lineages, such as *Myotis siligorensis alticraniatus*, which was split into *Myotis phanluongi* due to morphological and genetic divergence along an altitudinal gradient (Borisenko et al. 2009). These parallels highlight that altitudinal shifts, even subtle ones, can produce consistent structural differentiation in cranial modules.

#### Geographic variation in *G. c. commissarisi*

Geometric morphometrics of the parieto-occipital region, combined with LCA and wing analyses, reveal significant latitudinal variation in *G. c. commissarisi* (Tables 1 and 2). One Chiapas population at 16°N latitude differs from neighboring populations at 15°N and 17°N, indicating morphological differentiation across a narrow latitudinal gradient (Table 2).

This pattern aligns with the differentiation between the Veracruz Province and the Chiapas Highlands, reflecting the complex geological and biogeographical history of Nuclear Central America, a region characterised by environmental heterogeneity and topographic barriers that promote diversification (Rovito et al. 2015; Cano et al. 2018; Rodríguez-Gómez et al. 2021).

The latitudinal variation detected here may indicate local adaptation or early-stage divergence. Comparable shallow but structured variation has been reported in *Artibeus lituratus, Sturnira parvidens, Tadarida brasiliensis*, and other Neotropical bats (Marchán-Rivadeneira et al. 2011; Gonçalves et al. 2018; Torres-Morales et al. 2019). In *G. c. commissarisi*, shape differences are modest—as expected for a highly conserved basicranial module—but clearly structured rather than random, consistent with divergence driven by broad environmental gradients.

### Sexual dimorphism

Our analyses detected significant sexual dimorphism in mandibular shape and centroid size, consistent with previous findings in *Glossophaga* (*G. antillarum, G. mutica, G. soricina*, and *G. valens*; Calahorra-Oliart et al. 2021) and in other nectarivorous bats, including *Monophyllus redmani* (Mancina and Balseiro 2010). Webster (1993) similarly reported sexual size dimorphism in multiple *Glossophaga* species, with females exhibiting larger cranial dimensions.

Sexual dimorphism in mandibular morphology may reflect adaptive mechanisms to reduce intersexual competition (Shine 1989), with females of several *Glossophaga* species incorporating broader dietary items (Sánchez-Casas and Álvarez 2000). These patterns often relate to the increased energetic demands of reproduction (Racey and Entwistle 2000; Encarnação and Dietz 2006; Maucieri et al. 2021).

Linear wing measurements also showed larger dimensions in females—forearm and metacarpals, consistent with aerodynamic adjustments that reduce wing loading during pregnancy (de Camargo and Oliveira 2012; Wu et al. 2018; López-Cuamatzi 2024). Sexual segregation in roosting behavior in other nectarivorous bats (e.g., *G. antillarum*; Goodwin 1970) further supports the ecological basis of these morphological distinctions.

Together, these findings suggest that sex-specific differences in cranial and wing morphology in *G. commissarisi* represent adaptive responses to the mechanical and energetic pressures associated with reproduction and resource use.

## Conclusions

Our study identified significant variations in skull shape (fronto-maxillary) and size-shape (mandible) between the two subspecies. *G. c. commissarisi* was found to be slightly larger and exhibited greater morphological variability compared to *G. c. hespera*. Latitude-based analyses revealed distinct patterns, with individuals from Nuclear Central America (16°N) differing significantly from other populations of *G. c. commissarisi*. Within *G. c. hespera*, biogeographic analyses of linear cranial analysis identified two distinct groups: populations from the Northern Pacific Lowlands (PACNOR) region diverged from those in other biogeographic provinces, including the Sierra Madre del Sur (SMS), which originates in Jalisco.

Significant sexual dimorphism was observed, manifesting in differences in mandibular shape, size, and wing morphology. Geographical factors emerged as major drivers of morphological differences in *G. c. commissarisi*, suggesting that biogeographic and ecological factors may significantly contribute to understanding lineages within this species.

The observed morphological differences may have functional and ecological implications. Variation in cranial and mandibular shape could reflect differences in feeding strategies, bite mechanics, or efficiency in exploiting floral resources, while differences in wing morphology may be associated with flight performance, habitat use, or foraging behavior across regions. These patterns suggest that the detected morphological divergence is not merely statistical, but may represent adaptive responses to local ecological conditions, reinforcing the role of geography and environment in shaping phenotypic differentiation within *Glossophaga commissarisi*.

To achieve a more comprehensive understanding of these taxa, genetic data should complement morphological analyses. Additionally, increasing sample size and incorporating specimens from a broader range of regions would improve the robustness of the findings.

Although this study focuses on morphological variation, we acknowledge that molecular data are essential for fully resolving taxonomic boundaries and evolutionary relationships within *Glossophaga commissarisi*. Ongoing molecular analyses are currently being developed for both subspecies; however, their scope has been constrained by the availability and preservation of tissue samples for all specimens available in museums. Many of them are suitable for morphometric analyses and lack viable genetic material, limiting their inclusion in molecular datasets. Despite this limitation, morphological approaches provide an independent and informative line of evidence, particularly valuable in rare taxa where comprehensive genetic sampling is not yet feasible. The integration of morphological and molecular data remains a central objective of our broader research framework, and future studies incorporating expanded genetic sampling will be crucial to further evaluate lineage divergence within this complex.

## Supplementary Material

obag015_Supplemental_Files

## Data Availability

The photographs of the skulls used in this study are available at the Zenodo repository (Zenodo.org). DOI: 10.5281/zenodo.17743796
