# Supplementary material for: Intraspecific and Geographical Variation of Glossophaga commissarisi in Mexico: Morphological Approach
Source: Integr Org Biol. 2026 Apr 30;8(1):obag015. doi: 10.1093/iob/obag015 (PMC13168884; doi:10.1093/iob/obag015)
Supplement: obag015_Supplemental_Files [file obag015_supplemental_files.zip › 4. Table S2.pdf]

INTRASPECIFIC AND GEOGRAPHICAL VARIATION OF GLOSSOPHAGA COMMISSARISI THROUGHOUT ITS MEXICAN DISTRIBUTION: A MORPHOLOGICAL APROACH

SUPPLEMENTARY DATA TABLE S2. Metadata and measurements of analyzed specimens using linear morphology: Mammal collection at the Museo de Zoología Alfonso L. Herrera, Facultad de Ciencias(MZFC), Colección Nacional de Mamíferos at the Pabellón Nacional de la Biodiversidad (CNMA), Vertebrate scientific collection of the Departamento de Ecología and Recursos Naturales at the Centro Universitario de la Costa Sur (CV-DERN). Species *G. c. c.* = *G. c. commissarisi* , *G. c. h.* = *G. c. hespera* . Sex: M= Male, F= Female. MS= Mexican state. MBP= Mexican Biogeographic Province: PACNOR= Pacific Lowlands (North), PACSUR= Pacific Lowlands (South), VP= Veracruzan Province, CHH= Chiapas Highlands Province, BB= Balsas Basin Province, Elev.= Elevation (meters above sea level). Linear Cranial Analysis: GLS= Greatest lenght of the skull, MTRL= Maxillary toothrow length, RW= , MB= Mastoid breadth; Linear Wing Analysis: FA= Forearm, TH= Thumb, M3= Metacarpal in the third digit , M4= Metacarpal in the fourth digit , F1-D3= First phalanx in digit three, F2-D3= Second phalanges in digit three, F1-D4= First phalanx from digit four.

METADATA							MEASUREMENTS										
							Linear Cranial Analysis (LCA) - mm				Linear Wing Analysis (LWA) - mm						
**Collection**	**ID**	***Species***	**Sex**	**MS**	**MBP**	**Elev.**	**GLS**	**MTRL**	**RW**	**MB**	**FA**	**TH**	**M3**	**M4**	**F1-D3**	**F2-D3**	**F1-D4**
CNMA	5854	*G. c. h.*	M	Colima	PACNOR	505	20.86	8.8	5.8	9.2	34.58	6.74	31.15	29.17	13.16	15.37	9.78
CNMA	5855	*G. c. h.*	F	Colima	PACNOR	521	20	7.7	5.7	9.3	36.47	7.24	32.93	30.88	13.32	17.05	10.97
CNMA	5856	*G. c. h.*	F	Colima	PACNOR	521	-	-	-	-	35.63	8.02	31.17	29.4	14.81	14.78	10.19
CNMA	6132	*G. c. h.*	M	Colima	PACNOR	400	20.5	7.5	5.8	9.4	-	-	-	-	-	-	-
CNMA	6133	*G. c. h.*	F	Colima	PACNOR	400	19.9	7.2	5.8	9.2	-	-	-	-	-	-	-
CNMA	6134	*G. c. h.*	M	Colima	PACNOR	400	-	-	-	-	37.35	8.51	33.3	30.64	12.93	14.55	10.27
CNMA	6138	*G. c. h.*	F	Colima	PACNOR	400	-	-	-	-	36.43	6.62	32.78	29.7	12.24	14.48	9.28
CNMA	6139	*G. c. h.*	M	Colima	PACNOR	400	-	-	-	-	35.11	8.54	31.14	28.78	13.6	15.25	10.53
CNMA	6142	*G. c. h.*	M	Colima	PACNOR	400	-	-	-	-	37.76	7.59	36.47	33.73	13.54	14.92	11.62
CNMA	6143	*G. c. h.*	M	Colima	PACNOR	400	20.64	7.6	5.6	9	35.53	6.98	31.05	28.33	12.45	14.38	9.38
CNMA	6152	*G. c. h.*	M	Colima	PACNOR	400	-	-	-	-	35.13	8.36	31.56	29.95	13.34	15.56	10.6
CNMA	6343	*G. c. c.*	F	Chiapas	PACSUR	370	-	-	-	-	35.26	5.78	31.54	30.25	13.47	16.57	9.68
CNMA	6555	*G. c. c.*	M	Oaxaca	VP	370	-	-	-	-	-	-	-	-	-	-	-
CNMA	7461	*G. c. h.*	M	Colima	PACNOR	506	20.84	8	5.7	9.4	35.96	7.89	32.77	30.06	13.07	14.54	9.46
CNMA	7462	*G. c. h.*	M	Colima	PACNOR	506	-	-	-	-	35.66	8.05	30.96	28.99	12.96	14.48	9.73
CNMA	7463	*G. c. h.*	M	Colima	PACNOR	506	-	-	-	-	35.35	7.69	30.8	28.34	12.61	14.96	10.48
CNMA	7965	*G. c. c.*	F	Chiapas	CHH	723	-	-	-	-	34.22	7.46	35.15	33.08	12.59	14.79	12.29
CNMA	8394	*G. c. c.*	F	Oaxaca	BB	1557	-	-	-	-	-	-	-	-	-	-	-
CNMA	11945	*G. c. h.*	F	Jalisco	PACNOR	60	20.2	7.8	5.6	9.2	35.02	6.63	30.12	27.41	12.93	14.92	9.84
CNMA	13648	*G. c. h.*	M	Nayarit	PACNOR	2	-	-	-	-	35.78	5.75	31.8	28.48	13.38	15.3	10.26
CNMA	13649	*G. c. h.*	M	Nayarit	PACNOR	2	-	-	-	-	-	-	-	-	-	-	-
CNMA	13650	*G. c. h.*	F	Nayarit	PACNOR	2	-	-	-	-	36.24	7.07	33.05	30.47	13.38	15.74	10.33

CNMA	14017	*G. c. h.*	M	Jalisco	PACNOR	47	20.21	8.2	5.9	9	-	-	-	-	-	-	-
CNMA	14018	*G. c. h.*	M	Jalisco	PACNOR	47	-	-	-	-	37.05	6.54	32.45	29.26	12.84	14.86	10.02
CNMA	19137	*G. c. c.*	M	Chiapas	VP	152	-	-	-	-	34.46	7.56	30.31	28.41	12.29	14.84	10.17
CNMA	19163	*G. c. c.*	M	Chiapas	CHH	1416	-	-	-	-	34.86	6.6	30.4	27.63	11.99	14.28	9.24
CNMA	19166	*G. c. c.*	F	Chiapas	VP	151	19.78	7.6	6.1	9.7	33.93	8.99	30.47	27.62	12.45	14.12	9.69
CNMA	19167	*G. c. c.*	M	Chiapas	VP	151	20.05	7.3	5.8	9.6	-	-	-	-	-	-	-
CNMA	19168	*G. c. c.*	M	Chiapas	VP	151	-	-	-	-	35.91	8.13	29.72	27.32	12.41	14.87	9.8
CNMA	20288	*G. c. c.*	M	Chiapas	VP	142	-	-	-	-	32.6	5.62	30.88	27.99	12.11	15.04	8.98
CNMA	20289	*G. c. c.*	F	Chiapas	VP	144	-	-	-	-	35.59	7.09	31.87	30.11	13.19	15.3	10.1
CNMA	22210	*G. c. c.*	F	Chiapas	VP	809	-	-	-	-	31.5	6.7	31	30.5	13.19	13.91	8.18
CNMA	22211	*G. c. c.*	M	Chiapas	VP	132	-	-	-	-	34.24	7.45	31.19	27.38	11.95	14.22	9.37
CNMA	22212	*G. c. c.*	M	Chiapas	VP	809	20.23	8	6.3	9.6	35.63	7.57	29.2	29.2	12.55	15.72	9.47
CNMA	22213	*G. c. c.*	M	Chiapas	VP	809	-	-	-	-	33.56	7.31	32.69	30.04	12.19	15.44	9.29
CNMA	22776	*G. c. c.*	F	Chiapas	VP	809	-	-	-	-	34.46	6.86	30.33	29.21	12.43	15.73	9.6
CNMA	22779	*G. c. c.*	F	Chiapas	VP	821	20.3	7.7	6.3	9.9	37.05	8.76	32.71	29.23	13.31	15.97	10.14
CNMA	22780	*G. c. c.*	M	Chiapas	CHH	1020	-	-	-	-	34.39	6.96	30.79	28.48	13.22	14.35	10.21
CNMA	22783	*G. c. c.*	M	Chiapas	CHH	1287	-	-	-	-	33.6	5.46	29.46	27.48	12.6	13.84	9.54
CNMA	22929	*G. c. c.*	M	Chiapas	VP	152	-	-	-	-	35.16	7.51	30.3	27.62	12.89	15.02	10.41
CNMA	24419	*G. c. c.*	F	Chiapas	VP	156	-	-	-	-	36.55	7.76	32.41	30.67	13.56	14.81	10.42
CNMA	24420	*G. c. c.*	F	Chiapas	VP	156	-	-	-	-	35.69	7.28	31.39	30	13.06	15.09	10.29
CNMA	24421	*G. c. c.*	M	Chiapas	VP	156	-	-	-	-	35.64	7.01	31.14	27.93	12.55	14.59	9.87
CNMA	24422	*G. c. c.*	M	Chiapas	VP	156	20.5	8	5.9	9.3	35.83	8.56	31.27	29.18	12.72	15.01	10.28
CNMA	24424	*G. c. c.*	M	Chiapas	VP	154	-	-	-	-	34.25	7.15	31.68	29.9	12.92	15.11	9.45
CNMA	41228	*G. c. h.*	M	Colima	PACNOR	250	-	-	-	-	36.39	6.89	31.21	27.86	13.03	15.69	9.74
CNMA	15739	*G. c. h.*	F	Jalisco	SMS	580	-	-	-	-	36.54	6.42	30.92	29.64	12.92	15.61	9.86
CNMA	15748	*G. c. h.*	F	Jalisco	SMS	580	-	-	-	-	34.86	7.18	30.16	28.33	12.46	13.73	9.36
MZFC	ghc448	*G. c. h.*	F	Jalisco	SMS	1092	20.3	8.5	6.3	9.9	35.95	6.24	34.45	31.18	13.08	14.61	10.04
MZFC	ghc449	*G. c. h.*	M	Jalisco	SMS	1092	20.9	8.1	6.3	9.6	34.77	7.25	32.65	31.62	13.45	13.6	10.2
MZFC	ghc453	*G. c. h.*	M	Jalisco	SMS	1092	20.97	8.1	6.2	9.7	34.78	7.2	34.34	31.86	14.08	15.55	10.43
CV-DERN	JAL250	*G. c. h.*	M	Jalisco	SMS	510	-	-	-	-	35.5	7.1	30.95	29.23	12.56	15.85	9.81
CV-DERN	JAL558	*G. c. h.*	M	Jalisco	PACNOR	510	20	8.3	6.3	9.7	35.56	5.93	31.05	27.38	14.16	15.29	11.01
CV-DERN	JAL578	*G. c. h.*	F	Jalisco	PACNOR	510	19	7.4	5.8	9.3	35.09	6.81	31.54	28.33	13.3	14.67	9.71
CV-DERN	JAL672	*G. c. h.*	F	Jalisco	SMS	1162	-	-	-	-	35.95	7.2	28.85	28.69	13.07	14.67	10.91
MZFC	umsp 143	*G. c. c.*	F	Oaxaca	VP	270	20.4	8.1	6.4	10	34.5	8.64	34.9	33.84	12.8	15.24	8.7
MZFC	UMSP006	*G. c. c.*	F	Oaxaca	VP	150	-	-	-	-	34.61	8.44	33.82	30.32	15.1	16.14	10.17
MZFC	UMSP009	*G. c. c.*	F	Oaxaca	VP	150	-	-	-	-	36.04	7.85	36.5	32.78	13.18	14.53	10.72
MZFC	UMSP018	*G. c. c.*	F	Oaxaca	VP	150	21.47	9	6.3	10	35.38	8.63	34.13	31.56	13.79	15.83	10.87
MZFC	umsp099	*G. c. c.*	M	Oaxaca	VP	150	-	-	-	-	34.64	5.83	33.95	33.42	11.98	14.7	8.95

MZFC	umsp110	*G. c. c.*	M	Oaxaca	VP	270	-	-	-	-	32.29	6.6	34.34	30.24	13.63	15.31	9.29
MZFC	UMSP115	*G. c. c.*	F	Oaxaca	VP	270	20.5	8.1	5.8	9.2	34.18	7.43	32.73	30.22	13.57	15.13	10.28
MZFC	UMSP162	*G. c. c.*	F	Oaxaca	VP	270	-	-	-	-	33.37	7.39	33.35	31.99	13.07	16.1	10.32
MZFC	UMSP230	*G. c. c.*	M	Oaxaca	VP	270	21.4	8.3	6.5	10.7	36.36	7.63	36.01	32.69	11.71	15.01	9.91
MZFC	UMSP313	*G. c. c.*	F	Oaxaca	VP	270	20.6	8.3	6	9.9	35.4	8.66	34.07	32.92	13.78	14.14	9.91
MZFC	UMSP314	*G. c. c.*	F	Oaxaca	VP	270	21.2	8.1	6.2	9.8	36.28	7.69	35.82	30.93	13.05	15.58	9.85
