# Supplementary material for: Intraspecific and Geographical Variation of Glossophaga commissarisi in Mexico: Morphological Approach
Source: Integr Org Biol. 2026 Apr 30;8(1):obag015. doi: 10.1093/iob/obag015 (PMC13168884; doi:10.1093/iob/obag015)
Supplement: obag015_Supplemental_Files [file obag015_supplemental_files.zip › 4. Table S3.pdf]

INTRASPECIFIC AND GEOGRAPHICAL VARIATION OF GLOSSOPHAGA COMMISSARISI THROUGHOUT ITS MEXICAN DISTRIBUTION: A MORPHOLOGICAL APROACH

SUPPLEMENTARY DATA TABLE S3- Description of landmarks and semi-landmarks, including landmark types, positions, and names for each of the four studied cranial configurations

Frontal-maxillary region

This region was analyzed with a set of 12 landmarks. Landmarks 1-7 (Type 1 and 2) were anatomically distinguishable points comprising internal landmarks (1-5) and landmarks on the structure's edge (6-7). Landmarks 8-12 were used to contour the rostral slope (Type 3).

LM	Type	Description
1	I	Posteriormost alveolar border of the last upper molar
2	I	Posteriormost alveolar border of the last upper premolar
3	I	Posteriormost alveolar border of the upper canine
4	I	Uppermost alveolar border of the second incisive
5	I	Anteriormost alveolar border of the first incisive
6	II	Uppermost point of the nasal bone
7	II	Upper frontomaxillary point at the level of the last upper molar (landmark 1)
8-12	III	Semilandmarks: border of the rostral slope

Image

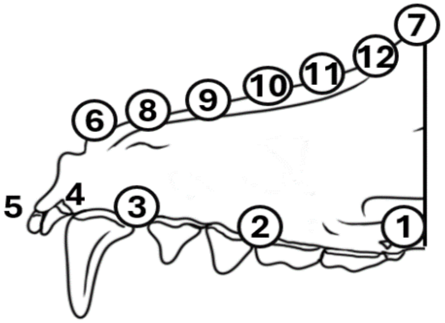

Parieto-occipital region

A total of 13 landmarks were used to characterize the parieto-occipital region. The first four points (1-4) correspond to anatomically distinguishable locations around the lateral view of the skull (Type II landmarks). Points 5-13 are semi-landmarks, outlining the entire sagittal crest (Type III landmarks).

LM	Type	Description
1	II	Upper frontomaxillary point at the level of the last upper molar
2	II	Anteriormost border of the foramen magnum
3	II	Posteriormost border of the foramen magnum
4	II	Extreme point at the angle at the most anterior end of the occipital condyle
5-13	III	Semilandmarks: contour of the sagittal crest

Image

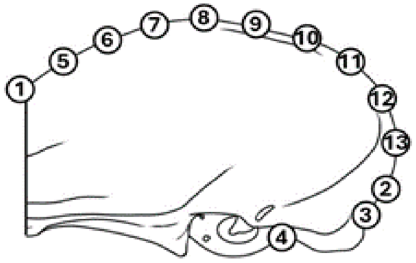

Mandible

In this configuration 19 landmarks were identified to analyze: Landmarks 1-7: were anatomically distinguishable landmarks. Points 1-5 are located on the structure's edge(Type 2), while points 6-7 are internal landmarks in the canine and premolar alveolar regions(Type 1). Points 8-19 were located as contour points (Type 3) describing the shape of the mandibular corpus.

LM	Type	Description	Image
1	II	Posteriormost alveolar border of the last lower molar	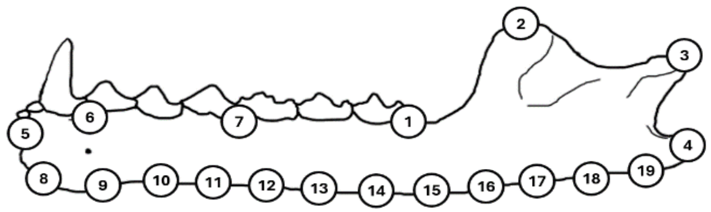
2	II	Point of maximum curvature at the coronoid process	
3	II	Upper margin of the condyle process	
4	II	Tip of the angular process	
5	II	Extreme point at the external anteriormost margin of the dentary bone at the base of the incisors	
6	I	Posteriormost alveolar border of the lower canine	
7	I	Posteriormost alveolar border of the last lower premolar	
8-19	III	Semilandmarks: outline of the margin of the mandibular corpus	

Ventral view of skull

Ten landmarks were analyzed in the ventral region, primarily consisting of internal landmarks (anatomically functional zones): Points 1-5 and 7: (Type 1). Point 6: Interdental zone on the structure's edge (Type 2). Points 8-10: Semilandmarks (Type 3) located on the procumbency of the lower incisor teeth, a highly variable region among *Glossophaga* species.

LM	Type	Description	Image
1	I	Anterior limit of the foramen magnum	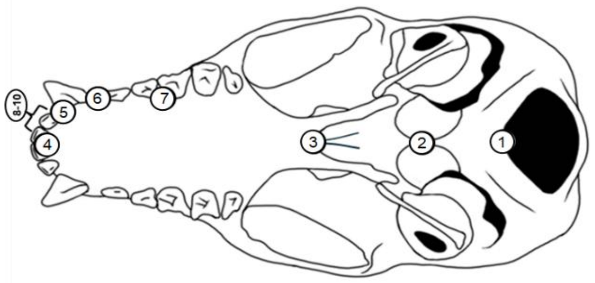
2	I	Baciooccipital sutura	
3	I	Palatine sutura	
4	I	Incisive fissure	
5	II	Mid point between canine and second incisor	
6	I	First upper premolar	
7	I	Mid point between last upper premolar and first molar	
8-10	III	Semilandmarks: Outline of the upper incisors procumbency	
