# Supplementary material for: Intraspecific and Geographical Variation of Glossophaga commissarisi in Mexico: Morphological Approach
Source: Integr Org Biol. 2026 Apr 30;8(1):obag015. doi: 10.1093/iob/obag015 (PMC13168884; doi:10.1093/iob/obag015)
Supplement: obag015_Supplemental_Files [file obag015_supplemental_files.zip › 4. Table S4.pdf]

INTRASPECIFIC AND GEOGRAPHICAL VARIATION OF GLOSSOPHAGA COMMISSARISI THROUGHOUT ITS MEXICAN DISTRIBUTION: A MORPHOLOGICAL APROACH

Table S4. —Summary of PCA results for each cranial configuration; eigenvalues, proportion of explained variance, and cumulative variance for PC1–PC6. Components PC7–PC34 pooled.

	Fronto-maxillary			Mandible		
	**Eigenvalue**	**Proportion**	**Cum. Prop**	**Eigenvalue**	**Proportion**	**Cum. Prop**
**PC1**	0.00067	0.33576	0.33576	0.00022	0.26459	0.26459
**PC2**	0.00061	0.30965	0.64541	0.00016	0.19526	0.45984
**PC3**	0.00028	0.13920	0.78461	0.00015	0.18828	0.64812
**PC4**	0.00012	0.05800	0.84260	0.00008	0.09104	0.73377
**PC5**	0.00010	0.05232	0.89493	0.00006	0.07392	0.80769
**PC6**	0.00004	0.02112	0.91604	0.00004	0.04727	0.85496
**PC7-34**	0.00017	0.08396	13.81617	0.00012	0.14509	21.51949

	Parieto-occipital			Ventral		
	**Eigenvalue**	**Proportion**	**Cum. Var**	**Eigenvalue**	**Proportion**	**Cum. Prop**
**PC1**	0.00034	0.39182	0.39182	0.00006	0.31757	0.31757
**PC2**	0.00019	0.21354	0.60553	0.00004	0.24496	0.56252
**PC3**	0.00013	0.15379	0.75915	0.00003	0.17221	0.73474
**PC4**	0.00008	0.09574	0.85489	0.00001	0.08216	0.81689
**PC5**	0.00003	0.03959	0.89448	0.00001	0.06553	0.88242
**PC6**	0.00003	0.03283	0.92730	0.00001	0.03848	0.92090
**PC7-34**	0.00002	0.02378	12.96032	0.00001	0.07843	9.86956
