# Supplementary material for: Intraspecific and Geographical Variation of Glossophaga commissarisi in Mexico: Morphological Approach
Source: Integr Org Biol. 2026 Apr 30;8(1):obag015. doi: 10.1093/iob/obag015 (PMC13168884; doi:10.1093/iob/obag015)
Supplement: obag015_Supplemental_Files [file obag015_supplemental_files.zip › 4. Table S5.pdf]

INTRASPECIFIC AND GEOGRAPHICAL VARIATION OF GLOSSOPHAGA COMMISSARISI THROUGHOUT ITS MEXICAN DISTRIBUTION: A MORPHOLOGICAL APROACH

Table S5. —Results of Procrustes ANOVA used to evaluate measurement error in the geometric morphometric analyses of four cranial configurations (ventral, parieto-occipital, mandible, and fronto-maxillary).

	Measurement Error Analysis				
	VENTRAL				
	**Rsq**	**F**	**Z**	**Pr(>F)**	**sample**
v_discrete	0.99939	1810.6846	2.56503	**0.001****	13 specimens
vrepetition	0.00005	1.1892	0.57292	0.324	
	PARIETO-OCCIPITAL				
	**Rsq**	**F**	**Z**	**Pr(>F)**	**sample**
v_discrete	0.99959	2654.8321	23.2646	**0.001****	30 specimens
vrepetition	0.00004	2.8033	1.3557	0.095	
	MANDIBLE				
	**Rsq**	**F**	**Z**	**Pr(>F)**	**sample**
v_discrete	0.99837	693.3108	9.173	**0.001****	29 specimens
vrepetition	0.00019	3.6118	1.5178	0.066	
	FRONTO-MAXILLARY				
	**Rsq**	**F**	**Z**	**Pr(>F)**	**sample**
v_discrete	0.99791	483.6445	14.5746	**0.001****	36 specimens
vrepetition	0.00002	0.4152	-0.2306	0.582	
