# Supplementary figures and images for: Intraspecific and Geographical Variation of Glossophaga commissarisi in Mexico: Morphological Approach
Source: Integr Org Biol. 2026 Apr 30;8(1):obag015. doi: 10.1093/iob/obag015 (PMC13168884; doi:10.1093/iob/obag015)

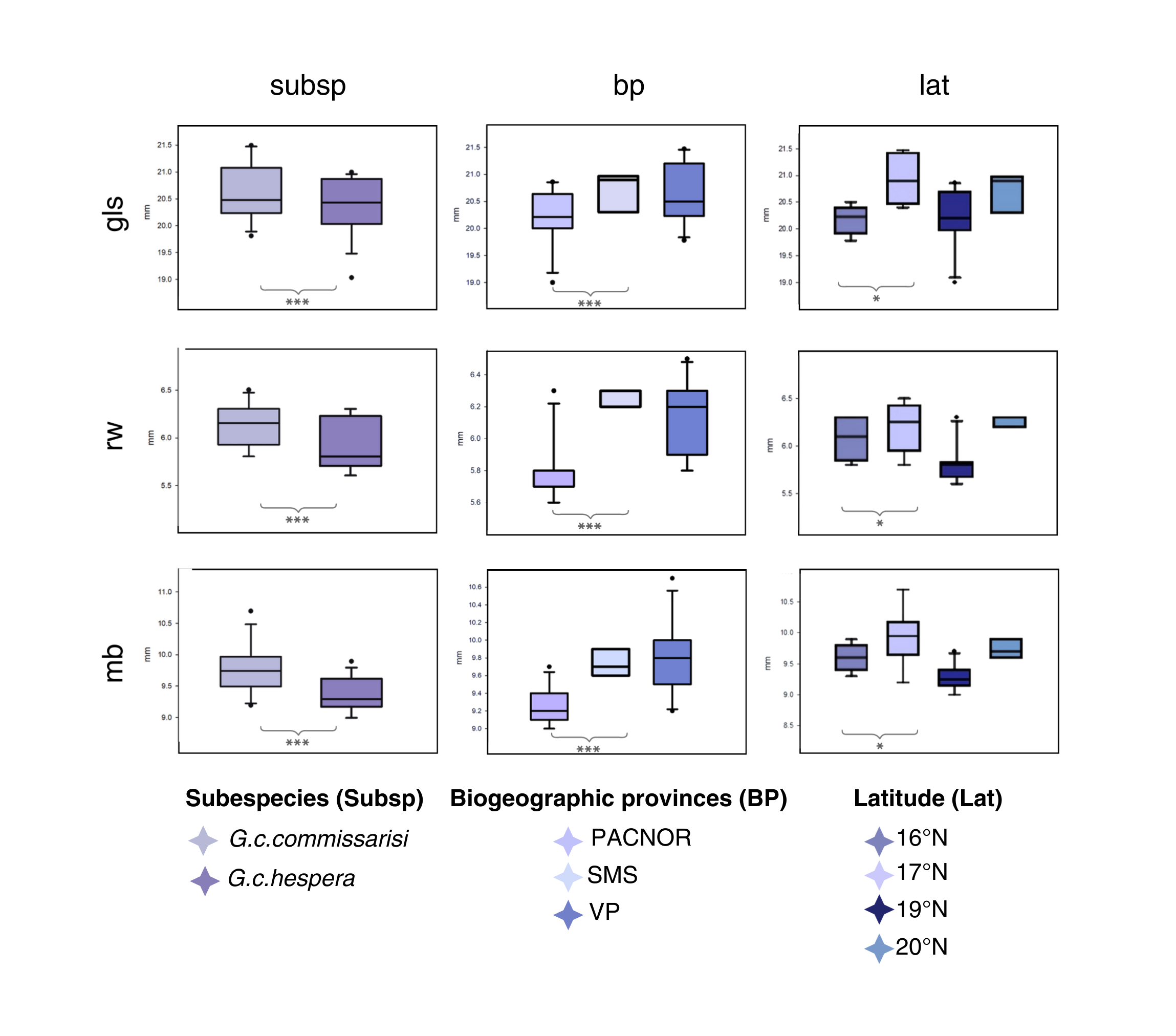

Supplement: obag015_Supplemental_Files [file obag015_supplemental_files.zip › 4. Fig S1.tif]

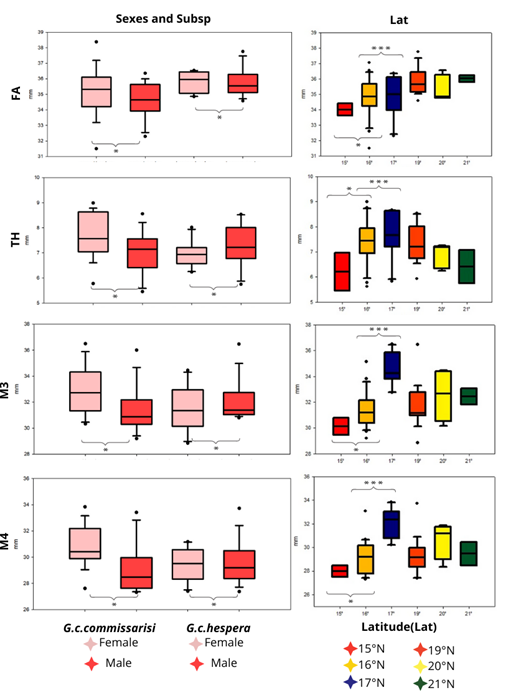

Supplement: obag015_Supplemental_Files [file obag015_supplemental_files.zip › 4. Fig S2.tif]

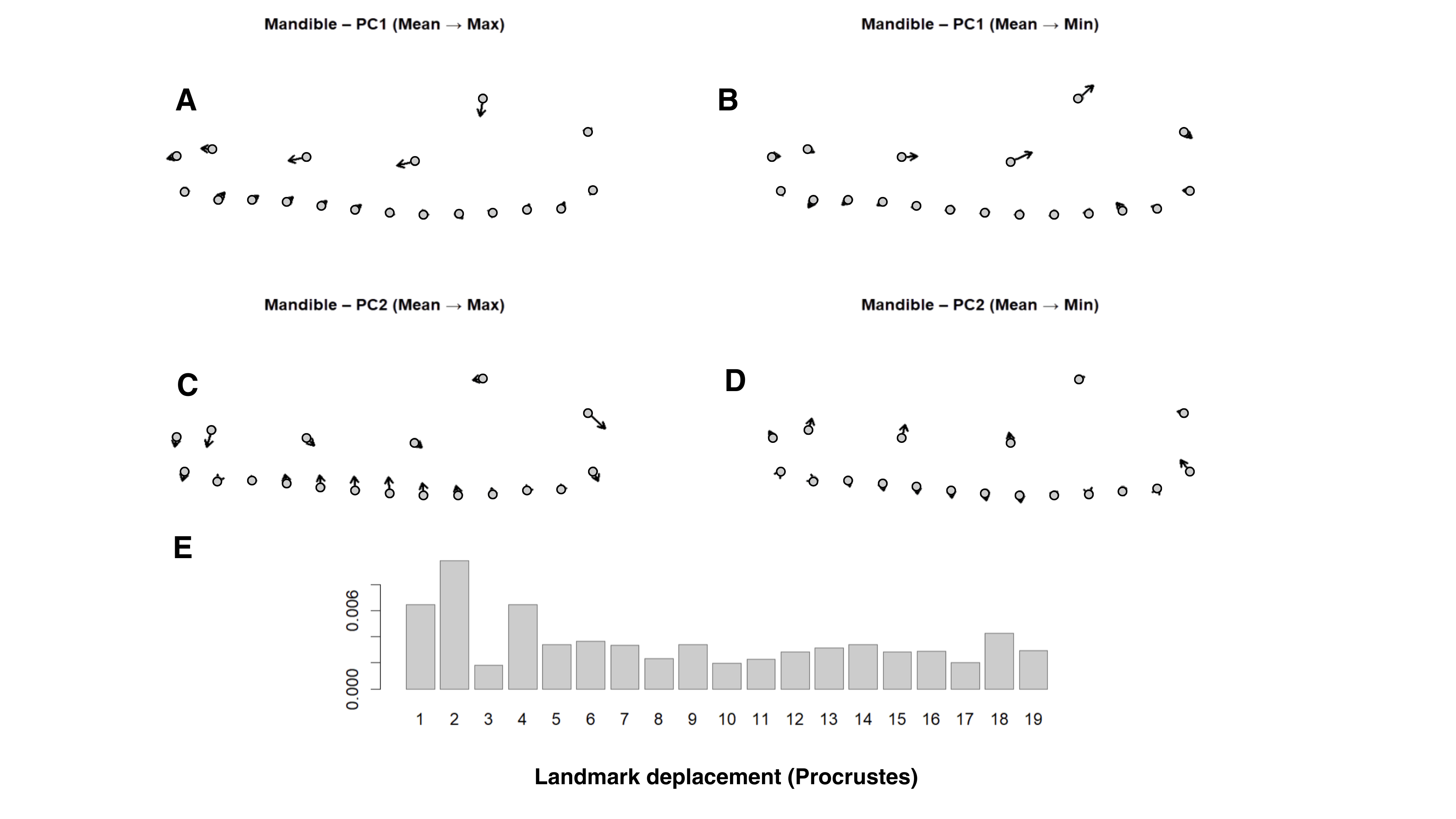

Supplement: obag015_Supplemental_Files [file obag015_supplemental_files.zip › 4. Fig S3.tif]
